# *Mucuna pruriens* Protects against MPTP Intoxicated Neuroinflammation in Parkinson’s Disease through NF-κB/pAKT Signaling Pathways

**DOI:** 10.3389/fnagi.2017.00421

**Published:** 2017-12-19

**Authors:** Sachchida N. Rai, Hareram Birla, Saumitra S. Singh, Walia Zahra, Ravishankar R. Patil, Jyoti P. Jadhav, Mallikarjuna R. Gedda, Surya P. Singh

**Affiliations:** ^1^Department of Biochemistry, Institute of Science, Banaras Hindu University, Varanasi, India; ^2^Department of Biotechnology, Shivaji University, Kolhapur, India

**Keywords:** Parkinson’s disease, neuroinflammation, *Mucuna pruriens*, tyrosine hydroxylase, MPTP (1-methyl-4-phenyl-1, 2, 3, 6-tetrahydropyridine), substantia nigra

## Abstract

Till date, drugs that have been used to manage Parkinson’s disease (PD) have only shown symptomatic relief with several adverse effects besides their inability to prevent neurodegeneration. Neuroinflammation plays an important role in the advancement of PD and can be targeted for its effective treatment. Researchers have suggested that herbal plants exhibiting the anti-inflammatory and anti-oxidant properties are therefore beneficial to human health. Conventionally, *Mucuna pruriens* (Mp) seeds are used for maintaining male virility in India. Reportedly, Mp is used as a rejuvenator drug having neuroprotective property. Our study aimed to investigate effects of aqueous extract of Mp (100 mg/kgbwt) on neuroinflammation, orally administered to mice intoxicated with 1-methyl-4-phenyl-1,2,3,6-tetrahydropyridine (MPTP) as well as the molecular mechanism involved in the progression of PD. In this study, we have observed significant behavioral abnormalities beside decreased antioxidant defense in MPTP intoxicated mice. We have also observed significant increase in inflammatory parameters like Glial Fibrillary Acidic Protein, Inducible Nitric Oxide Synthase, Intercellular Cell Adhesion Molecule, and Tumor Necrosis Factor alpha in substantia nigra pars compacta (SNpc) of parkinsonian mice, while Mp treatment has notably reduced these inflammatory parameters. Mp also inhibited the MPTP induced activation of NF-κB and promoted pAkt1 activity which further prevented the apoptosis of the dopaminergic neurons. Moreover, Mp exhibited significant antioxidant defense by inhibiting the lipid peroxidation and nitrite level, and by improving catalase activity and enhancing GSH level in nigrostriatal region of mouse brain. Mp also recovered the behavioral abnormalities in MPTP treated mice. Additionally, Mp treatment considerably increased the immunoreactivity of Tyrosine Hydroxylase and Dopamine Transporter in SNpc of parkinsonian mice. Our high performance liquid chromatography analysis of the Mp seed extract have shown L-DOPA, gallic acid, phytic acid, quercetin, and catechin equivalents as the major components which might cause neuroprotection in PD mice. Our result suggested that Mp extract treatment containing L-DOPA and a mixture of rich novel phytochemicals significantly alleviates the MPTP induced neurotoxicity by NF-κB and pAkt pathway. The findings observed thereby indicate that Mp extract have suggestively ameliorated MPTP induced neuroinflammation, restored the biochemical and behavioral abnormalities in PD mouse and thus provided a scientific basis for its traditional claim.

## Introduction

After Alzheimer’s disease (AD), Parkinson’s disease (PD) is considered to be the most common progressive neurodegenerative disease. PD is basically characterized by the loss of dopaminergic neurons in substantia nigra pars compacta (SNpc) and reduced level of dopamine (DA) within the striatum (ST) (Siderowf and Stern, 2003; Yadav et al., 2013, 2014; Rai et al., 2016). Among non-motor symptoms, cognitive decline appears the major one in the case of PD (Aarsland et al., 2017). Dopaminergic neuronal degradation, formation of inclusions called Lewy bodies and activation of glial cells are the hallmark of PD pathogenesis in brain. While the etiopathogenesis of PD still not fully known, it is identified to go worse on exposure to environmental neurotoxins such as MPTP, Paraquat (PQ), and several others (Bové et al., 2005; Khan et al., 2013; Yadav et al., 2013). In humans and primates, MPTP which is a potent inhibitor of mitochondrial complex-1 of electron transport chain creates parkinsonian characteristic, and in mice, it recapitulates dopaminergic degenerations via nigrostriatal pathway. For that reason, in animal models of PD, MPTP is extensively used to study and explore the molecular events responsible for dopaminergic neuronal degeneration and to check the efficacy of several neuroprotective agents (Jackson-Lewis et al., 2012). Biomolecules such as lipids, proteins, and DNA are damaged by reactive oxygen species (ROS) and reactive nitrogen species (RNS), by-products of which were observed in the SN and striatum of human PD post-mortem brains (Dexter et al., 1994; Khan et al., 2013). The oxidation of lipid and proteins can thus lead to loss of membrane integrity, enzyme inactivation leading to cell death in neurodegenerative disorders (Hald and Lotharius, 2005).

Previous literature suggests that in PD, prolonged neuroinflammation plays an important role during degeneration of neurons (Hirsch and Hunot, 2009; Joglar et al., 2009; Khan et al., 2013). Inflammatory response during neurodegeneration has not yet been thoroughly investigated. Proinflammatory mediators such as cytokines/chemokines, enzymes like cyclooxygenase-2 (COX-2) and inducible nitric oxide synthase (iNOS) are produced by glial (astroglial and microglial) cells in response to extracellular insult to dopaminergic neurons (Khan et al., 2013). In addition, nuclear transcription factor-κB (NF-κB) plays a central role in the PD pathogenesis by inducing the expression of tumor necrosis factor-alpha (TNF-α) and interleukin-1 beta (IL-1β) via oxidative stress mediated neurodegeneration (Hald and Lotharius, 2005). These cytokines and enzymes could cause neuronal death by the cytotoxic mechanism. Previous literature has also suggested that anti-inflammatory drugs significantly inhibit the neuroinflammatory processes and ultimately protect dopaminergic neuronal loss in different parkinsonian models (Choi et al., 2005; Jin et al., 2005).

In spite of important evidences in learning the pathobiology of neurodegeneration, various efforts to expand and advance successful treatment strategies are being done (Brotchie and Jenner, 2011) with minimal translational results. With this background, there has been a lot of focus on the herbal mediated neuroprotection of PD with special emphasis on the anti-oxidative and anti-inflammatory activities of these herbal plants and their derivatives. Recently, Kim et al. (2015) suggested that *Ligusticum officinale* exhibits potent anti-inflammatory activity via NF-κB/IκB-α and MAPK pathway. Pycnogenol, extracted from *Pinus maritima* bark also protects dopaminergic neuron in MPTP induced neuroinflammation (Khan et al., 2013).

In Indian system of medicine, *Mucuna pruriens* (Mp) is the most accepted drug. A number of reports have recommended that it exhibits various pharmacological properties like analgesic, anti-inflammatory, anti-neoplastic, anti-epileptic, and anti-microbial activities (Sathiyanarayanan and Arulmozhi, 2007; Adepoju and Odubena, 2009; Yadav et al., 2013). Mp has been found to be rich in bioactive compounds such as tannins, alkaloids, phenolics compounds, and flavonoids (Duke, 1995). Diabetes, atherosclerosis, rheumatoid arthritis, nervous disorders, and Parkinsonism are certain diseases that have been found to be effectively managed by free-radical mediated disease management property of Mp seeds (Bhaskar et al., 2011). Moreover, our high performance liquid chromatography (HPLC) data has shown the presence of different phytochemicals such as proanthocyanidin, tannin, gallic acid, quercetin, and phytic acid in the aqueous seed extract of Mp. The anti-neuroinflammatory activity of Mp might be due to the presence of these active constituents. Also, other phytochemicals can act in combination for exploring its synergistic effect. Recently, Uchegbu et al. (2016), have suggested the anti-inflammatory activity of Mp by administering the doses of 10 and 50 mg/kgbwt in carrageenan and formalin induced acute and chronic paw edema respectively. They compared the anti-inflammatory activity of Mp with diclofenac potassium as a standard anti-inflammatory drug. At the doses of 10 and 50 mg/kg, Mp showed an inhibition % of 9.8 and 47.8 and 6.6 and 38.8 respectively against their corresponding carrageenan and formalin induced acute paw edema (Uchegbu et al., 2016). Additionally, Yadav et al. (2017) showed that Mp seed extract (100 mg/kg bwt) has improved the neurobehavioral activity by reducing the oxidative stress in nigrostriatal tissue effectively by mitigating the iNOS expression levels in Paraquat (PQ) induced parkinsonian mouse model. Mp contains L-DOPA and ursolic acid which has potent anti-parkinsonian property (Rai et al., 2016; Yadav et al., 2016). There is very little literature which shows the anti-inflammatory activity of Mp in PD. With all the background information, in the present study, we have chosen Mp seed extract in MPTP induced Parkinsonian mouse model to explore its potent anti-neuroinflammatory activity.

## Materials and Methods

### Reagents and Antibodies

Acetic acid, disodium hydrogen phosphate, reduced glutathione (GSH), reduced nicotinamide adenine dinucleotide phosphate (NADPH), Potassium chloride, Ammonum chloride, Sodium dihydrogen phosphate, and Bovine Serum Albumin (BSA) were procured from Sisco Research Laboratories (SRL; Mumbai, India). 1-Methyl-4-phenyl-1,2,3,6-tetra hydropyridine (MPTP), Normal Goat Serum (NGS) from Sigma–Aldrich (St. Louis, MO, United States). Protein estimation kit by Bradford GeNeiTM, hydrogen peroxide (H_2_O_2_), and potassium dichromate were purchased from Merck (Darmstadt, Germany), Sodim dodecyl sulfate (SDS), Thiobarbituric acid (TBA), Griess reagent and DABCO were procured from HiMedia (Mumbai, India). Sodium nitrite and Paraformaldehyde were purchased from Lobachemie, India. Primary antibodies for TH (SC-25269), iNOS (SC-651), Glial Fibrillary Acidic Protein (GFAP) (SC-33673) and Intercellular Cell Adhesion Molecule (ICAM) (SC-8439) were procured from Santa Cruz, Biotechnology (Santa Cruz, CA, United States) and the primary antibodies for TNF-α (ab1793), NF-κB (ab16502), DAT (ab111468), and pAkt1 (ab81283) were purchased from Abcam Life Science, Biogenuix Medsystems, Pvt. Ltd. (New Delhi, India), secondary fluorescent tagged antibodies for IHCCy2-conjugated and cy3-conjugated were procured from Merck Millipore and Chemicon respectively.

### Experimental Animals

Eight to ten weeks old male mice (Swiss Albino mice, 25–30 g) were purchased from animal research facility of Banaras Hindu University, Varanasi, India. Before starting the experiment, animals were made to adapt the laboratory conditions for about a week under standard laboratory conditions by keeping light and dark cycles of 12 h. Mice were fed with standard rodent food purchased from market and water *ad libitum*. Experiments were done in between 12:00 noon to 03:00 pm. The investigational protocol for animals on which the test was carried out, was approved by the Animal Ethics Committee of Banaras Hindu University, Varanasi, India.

### Plant Extracts Preparation

Mp seed powder was purchased from the Ayurveda Pharmacy, Institute of Medical Sciences, Banaras Hindu University, Varanasi, India. Mp seed extract was prepared by the method of Uhegbu et al. (2005) in which distilled water was used as the solvent. 20 g of Mp seed powder was taken and soaked in 200 ml of autoclaved distilled water. The solution was stirred for about 6 min and left overnight for proper mixing. Next day, the solution was filtered by using filter paper (Whatman No. A-1) and the extract were allowed to dry in rotary vacuum evaporator under reduced pressure and temperature (below 40°C).

### RP-HPLC Quantitative Analysis of L-DOPA and Phytochemicals

L-DOPA was quantified in Mp seeds using reverse phase high performance liquid chromatography (RP-HPLC) involved with diode array detection. Samples were prepared as described by Rathod and Patel (2014) with minor modifications. One gram powder of Mp seed was extracted using autoclaved doubled distilled water: 0.1 M HCl (70:30) for 30 min on rotary shaker (120 rpm) and sonicated for 5 min. Then sample was evaporated, dissolved in distilled water and filtered through 0.45 μm nylon filter (Axiva filters). RP-HPLC analysis was performed by Shimadzu prominence equipped with degasser DGU-20A 5R, photo diode array detector SPD- M20 A and low pressure quaternary pump LC 20 AD. Chromatographic separation was achieved using a Waters, Nova-Pak C18 column (4 μm, 4.6 mm × 250 mm). The commercially available synthetic L-DOPA (Himedia) was taken as a standard.

Total phenolics content of Mp seeds has been determined spectrophotometrically (Singleton and Rossi, 1965). The sample was mixed with 1.8 ml of Folin–Ciocalteu reagent and incubated for 5 min at 25°C with 1.2 ml of 15% sodium carbonate solution for neutralization of reaction and further kept for 90 min at room temperature. The absorbance was taken at 765 nm. Result was noted in terms of mg of gallic acid equivalent per gram (mg GAE g^-1^) of dry mass.

Total flavonoids were quantified according to the method by Chang et al. (2002). In brief, 1 ml aqueous extract of Mp seed was added to 1.5 ml distilled water, 0.1 ml of aluminum chloride (10%) and 0.1 ml of potassium acetate (1 M). The total volume was made up to 4.5 ml by adding distilled water. Incubation was carried out for 30 min at room temperature and absorbance was recorded at 415 nm. Expression of flavonoids level was done as milligram of quercetin equivalents per gram (mg QUE g^-1^) of dry weight.

Evaluation of proanthocyanidin was done as described by Sun et al. (1998). The 0.5 ml sample was mixed with 3 ml of 4% vanillin and 1.5 ml of concentration HCl. Reaction mixture was incubated for 15 min and absorbance was taken at 490 nm. Content of Proanthocyanidin was expressed as catechin equivalents per gram (mg CAE g-1) of dry weight.

As described by Kirk and Sawyer (1998), Tannin level was measured with the help of Folin–Denis colorimetric method. 5 ml Mp seed extract was mixed with 1 ml Folin–Ciocalteu reagent and 2.5 ml saturated sodium carbonate. The solution was further incubated for 90 min at 28°C after the final volume was made up to 50 ml. The color intensity was measured at 760 nm.

The content of phytic acid was estimated according to the method of Gao et al. (2007). In short, 1 ml of Mp seed extract was added with 1 ml volume of Wade reagent (0.03% FeCl_3_.6 H_2_O and 0.3% sulfosalicylic acid in D/W). Vortexing of the solution was done for 5 s and then it was put to centrifugation for about 10 min. Absorbance of supernatant was taken at 500 nm using UV-spectrophotometer.

### Experimental Design

The first group, i.e., control (*n* = 6) was treated with normal saline (i.p.). MPTP (30 mg/kg body weight) was prepared by dissolving it in 0.9% saline. The mice were injected (i.p.) twice with MPTP (30 mg/kg body weight) within 16 h of interval to induce PD to the second group (*n* = 6) (Yadav et al., 2014). The third group (*n* = 6) was first given two injections (i.p.) of MPTP (30 mg/kg body weight) within 16 h of interval and then daily orally treated with Mp seed aqueous extract (100 mg/kg body weight) from next day till 21 days after second MPTP injection.

Group I: Mice (*n* = 6) were given intraperitoneal (i.p.) injections of saline (0.9%), this served as control.

Group II: Mice (*n* = 6) were administered i.p. injections of MPTP (30 mg/kg body wt.), twice within 16 h interval.

Group III: Mice (*n* = 6) were first intoxicated (i.p.) with MPTP (30 mg/kg body wt.), twice within 16 h interval and from the next day they were orally treated with aqueous seed extract of Mp (100 mg/kg body wt.) daily for 21 days.

### Neurobehavioral Studies

#### Rotarod Test

In Rotarod test, prior to experiment, group animals were trained for 3 successive days at a fixed speed (5 rpm) and the time was noted after the mice fall up to a maximum of 5 min. The experiment was repeated four times for each animal; finally average time was calculated as described previously (Manna et al., 2006). The same procedure was repeated once the treatment was completed and the time taken by the mice to fall was noted down.

#### Hanging Test

In this test, mice were placed on a horizontal grid and allowed to have grip on it. This grid was then made upside down so that mouse hangs downward, gripping on it, until they lose their control and fall down. The experiment was repeated three times, and the hanging time was noted for each group (Mohanasundari et al., 2006).

#### Narrow Beam Walking Test

Motor coordination in mice was assessed using this test. Animals from each group were trained to move on stationary wooden narrow flat beam (1 cm) which was positioned at a height of 100 cm above the floor (L 100 cm × W 1 cm). Then according to Pisa, time spent in walking from one end of the beam to another was noted and the procedure was repeated thrice for each group’s animals (Pisa, 1998).

### Sample Preparation for Biochemical Studies

After completion of experiment, the animals were sacrificed by cervical decapitation from each groups (*n* = 3), the collection of nigrostriatal tissue was done individually and they were further homogenized in KCl buffer (Tris-HCl 10 mM, NaCl 140 mM, KCl 300 mM, ethylenediaminetetraacetic acid 1 mM, Triton-X 100 0.5%) at pH 8.0 complemented with phosphatase and protease inhibitor. Centrifugation of the tissue homogenates was done at 12,000 *g* at a temperature of 4°C for about 20 min for the estimation of antioxidant enzymes and different biochemical parameters.

### Biochemical Test

#### Catalase and Nitrite Test

In accordance with the decomposition of hydrogen peroxide, the Catalase activity was estimated (Kumar et al., 2010). Briefly 10% w/v tissue homogenate was added in phosphate buffer pH-7, distilled water, hydrogen peroxide (0.02 M) and incubated at room temperature for 1 min then potassium dichromate and acetic acid (1:3) solution was added and solution was allowed to boil for 15 min in boiling water bath and absorbance was taken at 570 nm. The activity of enzyme was measured in nmoles/min/mg protein.

By using standard procedure, Nitrite level was estimated in the supernatant (Granger et al., 1996). Supernatant of 10% w/v tissue homogenate was taken and ammonum chloride (0.7 mM) mixed with Griess reagent (0.1% *N*-naphthyl ethylenediamine and 1% sulfanilamide in 2.5% phosphoric acid) was added. The solution was allowed to stand at 37°C for 30 min, and the supernatant was then taken out to record the absorbance at 540 nm. By using the standard curve for sodium nitrite (10–100 μM), the total content of nitrite was calculated in terms of μmoles/mL.

### Lipid Peroxidation and GSH Test

Estimation of Lipid peroxidation was done in the same way as described previously (Ohkawa et al., 1979) with fewer modifications in the nigrostriatal tissue of the mouse brain. Briefly, for measuring the concentration of malondialdehyde (MDA), a reaction mixture containing 10% tissue homogenate (0.1 mL) was added in 10% SDS solution (0.1 mL) and was kept at room temperature for 5 min. After that, 20% acetic acid (0.6 mL) was added and the solution was incubated 2–5 min. At last 0.8% Thio-barbituric acid TBA (0.6 mL) was added and the solution was kept in a boiling water bath for 1 h. The reaction mixture was then allowed to cool, centrifugation was done and absorbance of the supernatant was taken at 532 nm against control. Expression of LPO levels was done as nano moles MDA/mg protein. Glutathione reductase (GSH) level in the brain homogenate was measured by the method described previously (Moron et al., 1979) and reported as μM GSH/mg tissue.

### Immunofluorescence Staining of TH, NF-κB, TNF-α, DAT, iNOS, GFAP, ICAM, and pAkt1 in SNpc

In the SNpc of brain, Immunofluorescence staining of TH, NF-κB, TNF-α, DAT, iNOS, GFAP, ICAM, and pAkt1 in SNpc was performed (Gorbatyuk et al., 2008). Mice were anesthetized with pentobarbital and the perfusion was done with 4% paraformaldehyde and the brains were post-fixed and collected. Using a cryomicrotome, the brain was cut in 25 μ thick coronal sections at the SN level (Leica, Wetzlar, Germany). Washing of the sections were done twice with 0.01 M PBS at pH 7.4 and then they were allowed to incubate with blocking reagent (10% normal goat serum in PBS, 0.3% Triton-X 100) for 1 h. Incubation of the sections with primary antibodies at 1:1000 dilutions was done with the polyclonal anti-mouse against TH, polyclonal anti-rabbit against NF-κB p65, monoclonal anti-mouse against TNF-α, polyclonal anti-rabbit against DAT, polyclonal anti-rabbit against iNOS, monoclonal anti-mouse against GFAP, monoclonal anti-mouse against ICAM, and monoclonal anti-rabbit against pAkt1 for 16 h at 4°C. The washing of the sections was again done five times in PBST and they were further incubated with Cy2-conjugated (Ex max 492 nm and Em max 510 nm) donkey anti-mouse and cy3-conjugated secondary antibodies (Ex max 550 nm and Em max 570 nm) donkey anti-rabbit in 1% BSA blocking solution for 1 h at Room Temperature. Washing of the sections was again done for three times and then mounted using mounting media, fluoro shield (Sigma–Aldrich). The images of the sections were taken with the help of fluorescent microscope Nikon (Thermo Fisher Scientific). Immunofluorescence was analyzed by Image J software (NIH, United States) and reported in mean integrated fluorescent value (IFV).

### Statistical Analysis

Statistical analysis of differences between means of groups was determined by one way ANOVA followed by Student-Newman–Keuls *post hoc* test using GraphPad Prism 7.0 software. A *p* < 0.05 was considered statistically significant.

## Results

### L-DOPA and Phytochemicals Content

**FIGURE 1:**
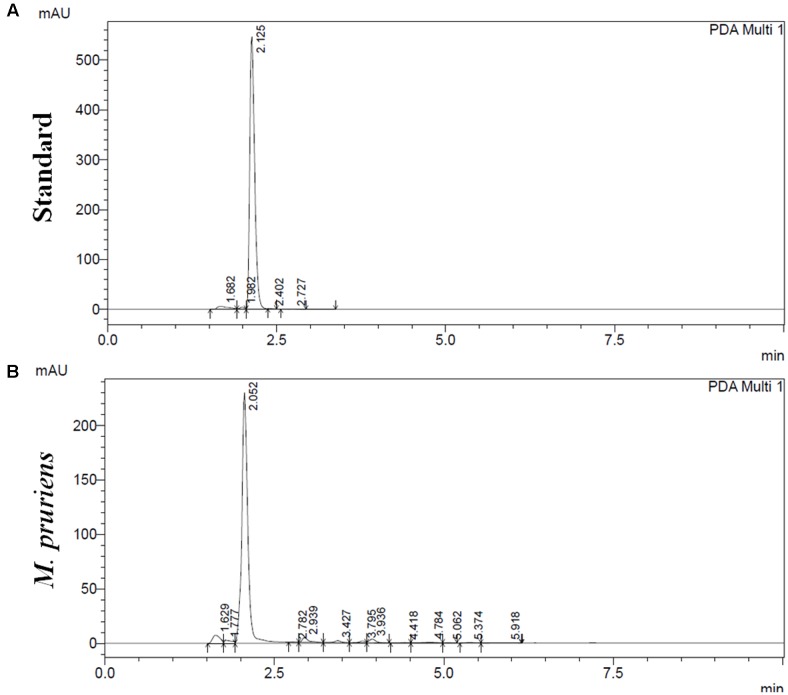
High performance liquid chromatography (HPLC) analysis of *M. pruriens* seeds for L-DOPA. HPLC profile of standard L-DOPA **(A)**. HPLC profile of *M. pruriens* seed extract **(B)**.

Reverse phase high performance liquid chromatography analysis showed as compared to standard L-DOPA (**Figure 1A**), 65 mg g^-1^ of L-DOPA present in the aqueous extract of Mp seed (**Figure 1B**). Mp showed 38.9 ± 1.6 mg per gram of gallic acid equivalent (mg GAE g^-1^) of phenolics and 54.14 ± 3.05 mg per gm of quercetin equivalent (mg QAE g^-1^). The Mp seed showed 25.71 ± 4.13 mg per gram of catechin equivalents (mg CAE g^-1^) proanthocyanidin level. Mp also showed 8.2 ± 0.15 mg g^-1^ of tannin level. Mp seed contain 6.72 ± 0.11 mg g^-1^ phytic acid.

### Behavioral Studies

#### Effect of Mp on Behavioral Recovery

**FIGURE 2:**
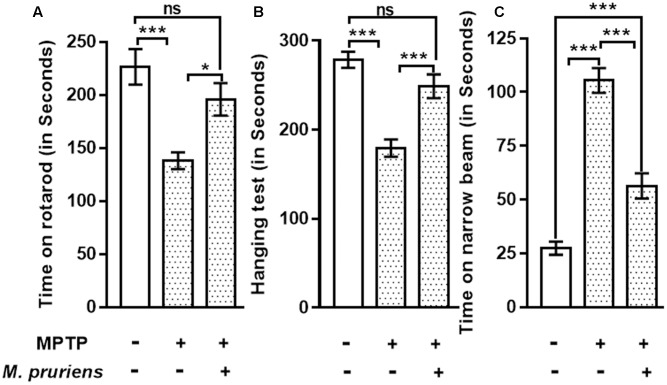
Effect of Mp extract on behavioral parameters. **(A)** MPTP PD mice showed significantly reduced time of walking and staying on rotarod as compared to CONT group, which has been significantly improved on Mp supplementation to PD mice in comparison with untreated PD mice. **(B)** Hanging test showed significant improvement in Mp treated group compared to MPTP treated group. MPTP group mice fall early as compared to CONT group. **(C)** Narrow beam walking time was significantly increased in the MPTP treated mice in comparison with control group while Mp treatment declines the narrow beam walking time as compared to MPTP induced PD mouse (^∗^*p* < 0.05, ^∗∗^*p* < 0.01, ^∗∗∗^*p* < 0.001, *n* = 6). ns, non-significant.

The result shows that the time taken by the MPTP treated mice for which it remained on the rotarod was significantly reduced (*p* < 0.001) compared to control. Whereas, when MPTP treated mice were treated with Mp, mice stayed on the rotarod significantly longer than MPTP mice (*p* < 0.05) (**Figure 2A**).

Our findings suggested that, in MPTP-treated mice, the time of gripping and hanging was significantly poorer (*p* < 0.001) as compared to control mice. When MPTP mice were treated with Mp, the hanging time was increased (*p* < 0.001) when compared with MPTP treated mice (**Figure 2B**).

Result shows that in MPTP treated mice narrow beam walking time was increased (*p* < 0.001) as compared to CONT mice. When MPTP mice were treated with Mp the narrow beam walk time was decreased (*p* < 0.001) as compared to MPTP mice (**Figure 2C**).

### Biochemical Studies

#### Effect of Mp on Catalase and Nitrite

**FIGURE 3:**
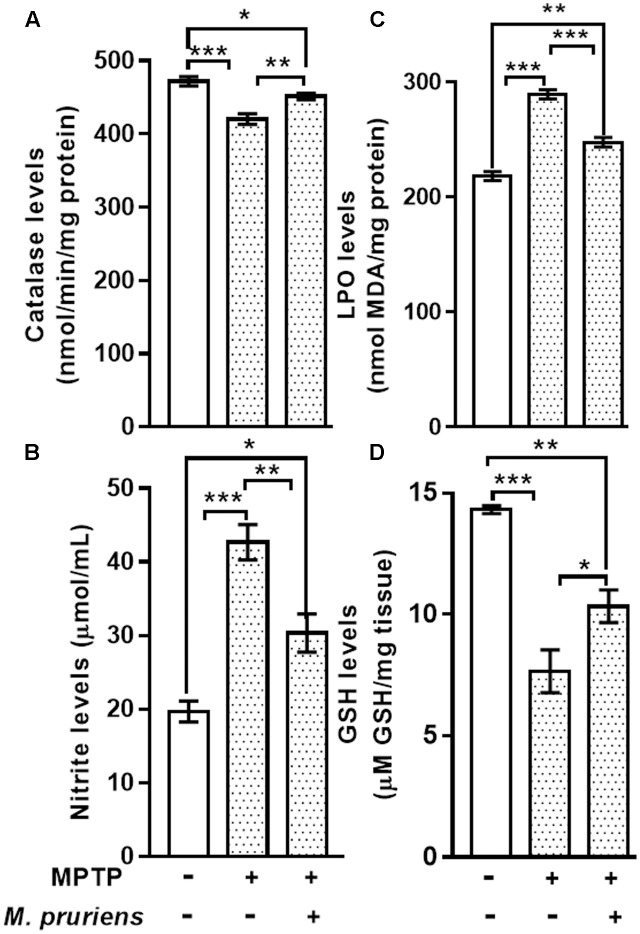
Estimation of CAT, Nitrite, MDA, and GSH in nigrostriatal region of mice. MPTP injected mice showed significant decrease in CAT activity and GSH level and increase in Nitrite and MDA level as compared to control (CONT) group. While Mp treatment in MPTP group significantly increase the level of CAT **(A)**, decrease in the level of nitrite **(B)** and MDA **(C)**, increase in the level of GSH **(D)**. Values are expressed as mean ± SEM (^∗^*p* < 0.05, ^∗∗^*p* < 0.01, ^∗∗∗^*p* < 0.001, *n* = 3). ns, non-significant; MDA, malondialdehyde; GSH, glutathione; CAT, catalase; SEM, standard error of mean.

We observed that MPTP injection significantly decrease the activity of CAT (*p* < 0.001) and increase the nitrite (*p* < 0.001) content in MPTP-injected mice when compared to CONT group. However, Mp treatment (MPTP+Mp) increased the activity of catalase (*p* < 0.01) (**Figure 3A**) and decreased nitrite level (*p* < 0.01) (**Figure 3B**) as compared to MPTP-treated group.

### Effect of Mp on MDA and GSH Content

When compared to CONT group, the mice intoxicated with MPTP showed a significant increment (*p* < 0.001) in lipid peroxidation product, known as MDA. Conversely, MPTP administration caused a significant decline in GSH (*p* < 0.001) levels when compared with CONT group. Mp treatment (MPTP+Mp) significantly attenuated (*p* < 0.001) the rise in MDA level (**Figure 3C**) and improved (*p* < 0.05) the GSH levels (**Figure 3D**) compared to the MPTP group.

### Effect of Mp on the Expression of TH, NF-κB, TNF-α, DAT, iNOS, GFAP, ICAM, and pAkt1 in SNpc

**FIGURE 4:**
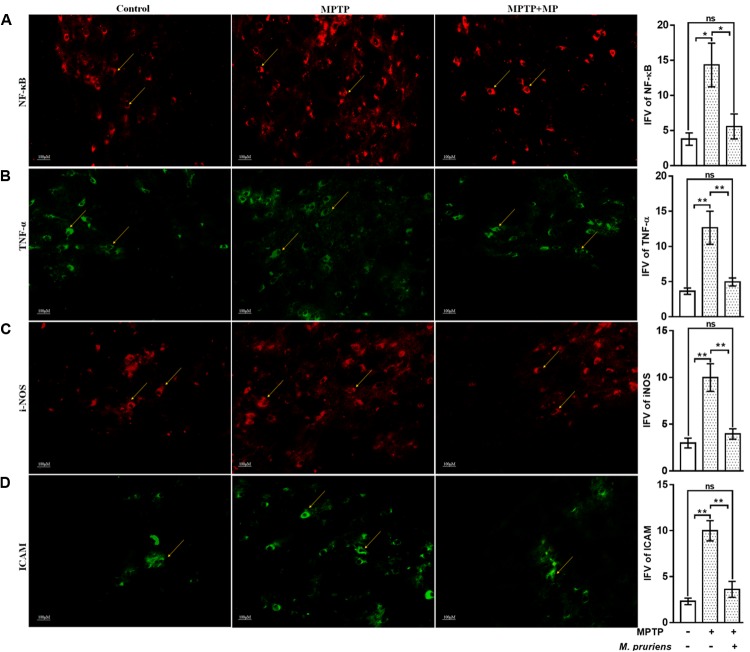
Immunofluorescence expression of NF-κB, TNF-α, iNOS, and ICAM in SNpc. of CONT, MPTP, and MPTP+Mp mice by using Image J Software at 20x magnification. The MPTP intoxicated PD mice showed significantly enhanced expression level of NF-κB **(A)**, TNF-α **(B)**, iNOS **(C)**, and ICAM **(D)** positive cells as compared to control. On Mp supplementation in PD mice showed significantly alleviated expression level of NF-κB, TNF-α, iNOS, and ICAM positive cells as compared to MPTP mice. Values are expressed as mean ± SEM of integrated fluorescent value (IFV) (^∗^*p* < 0.05, ^∗∗^*p* < 0.01, *n* = 3). ns, non-significant; TNF-α, tumor necrosis factor alpha; iNOS, inducible nitric oxide synthase; ICAM, intercellular adhesion molecule 1; SNpc, substantia nigra pars compacta.

**FIGURE 5:**
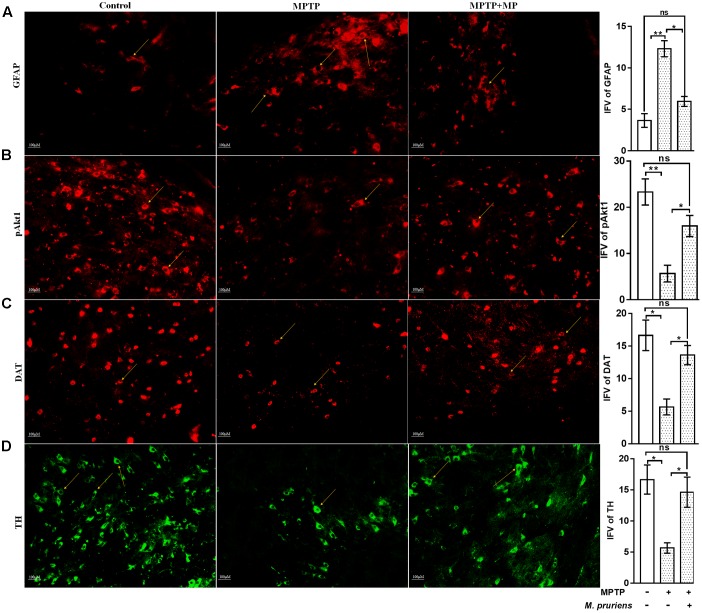
Immunofluorescence expression of GFAP, pAkt1, DAT, and TH in SNpc. of CONT, MPTP, and MPTP+Mp mice by using Image J Software at 20x magnification. The MPTP intoxicated PD mice showed significantly enhanced expression level of GFAP positive cells **(A)** as compared to control, while on Mp supplementation in PD mice showed significantly alleviated expression level of GFAP positive cells as compared to MPTP mice. On the contrary immunofluorescence staining of pAkt1 positive cells were reduced in MPTP treated mice **(B)** while Mp treatment substantially enhanced the expression of pAkt1. Similarly, DAT **(C)** and TH **(D)** positive dopaminergic neurons expression were reduced in MPTP treated mice while Mp treatment in MPTP treated mice significantly enhanced the expression of DAT and TH. Values are expressed as mean ± SEM of IFV (^∗^*p* < 0.05, ^∗∗^*p* < 0.01, *n* = 3). ns, non-significant; GFAP, glial fibrillary acidic protein; DAT, dopamine transporter; TH, tyrosine hydroxylase.

We have observed an increased in NF-κB (*p* < 0.05), TNF-α (*p* < 0.01), iNOS (*p* < 0.01), ICAM (*p* < 0.01), and GFAP (*p* < 0.01) positive cells expression in the MPTP treated mice as compared to CONT group. After Mp treatment (MPTP+Mp), a decrease in NF-κB (*p* < 0.05) (**Figure 4A**), TNF-α (*p* < 0.01) (**Figure 4B**), iNOS (*p* < 0.01) (**Figure 4C**), ICAM (*p* < 0.01) (**Figure 4D**), and GFAP (*p* < 0.05) (**Figure 5A**) expression was observed as compared to MPTP-treated mice. We also found decreased expression of pAkt1 positive cells (*p* < 0.01, **Supplementary Figure S1**) and DAT positive dopaminergic neurons (*p* < 0.05) as compared to control while Mp treatment (MPTP+Mp) significantly increased the expression of pAkt1 (*p* < 0.05) (**Figure 5B**) and DAT (*p* < 0.05) (**Figure 5C**) as compared to MPTP treated groups. A reduced (*p* < 0.05) level of TH positive dopaminergic neurons was seen in response to MPTP injection while comparing to the CONT group. However, following treatment with Mp in MPTP-administered mice, an increase in TH level (*p* < 0.05) (**Figure 5D**) was observed when compared to MPTP-treated mice.

### Effect of Mp on the Nuclear Translocation of NF-κB in SNpc

**FIGURE 6:**
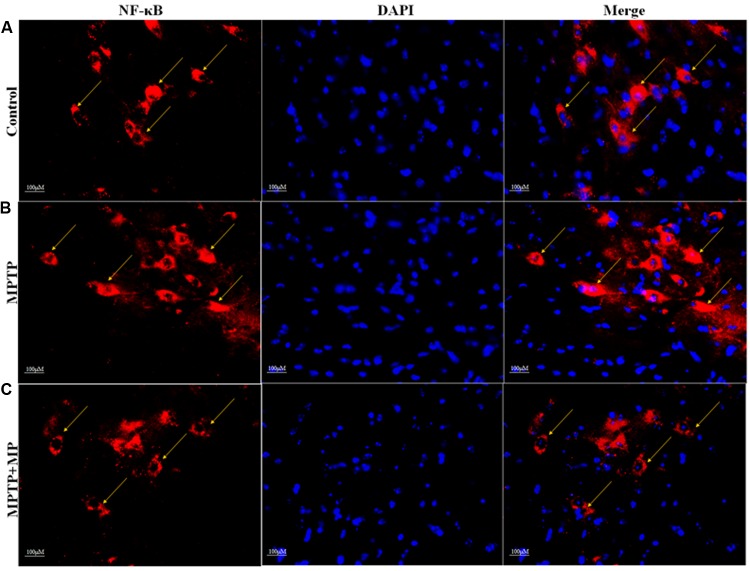
Effect of Mp on the nuclear translocation of NF-κB in SNpc with 40x magnifications after staining. In substantia nigra (SN) as compared to control group **(A)**, nuclear translocation of NF-κB positive cells were increased in MPTP treated group **(B)** while Mp treatment inhibits this nuclear translocation of NF-κB **(C)**.

We have observed that in MPTP injected mice, the nuclear translocation of NF-κB has occurred (**Figure 6B**) when compared with the control group (**Figure 6A**). Whereas, Mp treatment (**Figure 6C**) has significantly inhibited this nuclear translocation of NF-κB as compared to MPTP treated mice.

## Discussion

In this study we demonstrate that Mp, an aqueous extract containing L-DOPA and a mixture of rich novel phytochemicals protect nigrostriatal degeneration of dopaminergic neurons by alleviating oxidative stress and neuroinflammation in PD mouse model. Dopaminergic neurons can be protected by neuroinflammation linked inhibition of iNOS, GFAP, ICAM as well as NF-κB activation and its responsive genes TNF-α. Protective effect of Mp on dopaminergic neurons suggest that it is an efficient herbal agent in PD research, corroborating previous studies (Yadav et al., 2013, 2014, 2017; Olson and Gendelman, 2016). From several decades there is a lot of focus on Mp’s anti-parkinsonian activity specifically related to its anti-oxidative and metal chelating activity (Tharakan et al., 2007; Dhanasekaran et al., 2008; Yadav et al., 2013). Other than showing the anti-parkinsonian activity, Mp extract has also shown therapeutic potential in protecting against stroke and ischemia (Nayak et al., 2017). Mp extracts thus due to the presence of dopamine and 5-HT (5-hydroxytryptamine) has the potential to be an anti-cataleptic and antiepileptic drug (Champatisingh et al., 2011). *Ginkgo Biloba* extract 761 (EGb 761) is a well-defined mixture containing flavonoids (24%) and terpenoids (6%) and is patented (Rojas et al., 2009). This extract having the antioxidant and anti-apoptotic properties has the potential to show neuroprotection as it can help in the regulation of MAO. Phytomix-40 (PM-40) is a certified parapharmaceutical comprises extract from 40 plants including ginseng, eleutherococcus, *Rhodiola rosea* etc., which helps in improving hormonal, antioxidant and immune system of body. Administration of PM-40 along with standard anti- Parkinson’s drugs improves Parkinson’s symptoms and helps in decreasing negative activation of immune system which occurs during standard anti-Parkinson’s therapy alone (Bocharov et al., 2010). Anti-Parkinson’s activities of flavonoids have been also reported on 6-OHDA-induced experimental Parkinsonism *in vivo* and *in vitro* (Mu et al., 2009). Phenolics and flavonoids are important class of natural antioxidant substances having potential of scavenging free radicals, ultimately reducing the risk of oxidative stress related disorders including cancer and PD (Saxena et al., 2012; Patil et al., 2015). Recently, Cilia et al. (2017) have reported that 12.5–17.5 mg/kg Mp seed powder shows neuroprotective activity in PD patient with a more favorable tolerability profile.

The neuroprotective role of Mp in other neurodegenerative diseases such as AD and Multiple Sclerosis has not yet been investigated.

In this paper, we have reported the potential anti-neuroinflammatory activity of Mp in MPTP induced parkinsonian mouse model via NF-κB and Akt pathway.

Natural compounds containing flavonoids, phytochemicals having antioxidative and anti-inflammatory activities are neuroprotective in neurodegenerative diseases (Parkinson’s disease, 2012). Past studies potentially exhibiting that orally administered Mp reaches to the brain in adequate quantity to protect the dopaminergic neurons in PD (Katzenschlager et al., 2004; Yadav et al., 2013, 2014, 2017). To investigate the supplementary outcome of the drugs, oral route has been utilized as an efficient way of administration as it is the feasible and efficient way for drug delivery.

This study has demonstrated that in mice, MPTP intoxication creates behavioral impairment which is tested by rotarod, grip strength, and narrow beam walking test. Mp was found to significantly improve the motor deficits in parkinsonian mice. Our behavioral findings are in agreement with the earlier reports (Yadav et al., 2013, 2014).

Researchers have suggested that MPTP intoxication generates ROS and RNS which induces oxidative stress and neuroinflammation associated nigrostriatal degeneration of dopaminergic neurons (Yokoyama et al., 2008; Chung et al., 2011; More et al., 2013). Despite of the fact that, PQ poorly crosses the BBB, the patients died of PQ intoxication has been detected with significant damage to the brain (Bové et al., 2005). MPTP intoxication has helped in mimicking most of the parkinsonian symptoms which can help us in understanding PD (Meredith and Rademacher, 2011). MPTP induced Parkinsonian Mouse models have been most widely used. MPTP, being lipophilic in nature crosses the BBB easily and binds mainly in astrocyte lysosomes, where it is converted to its toxic metabolite, the 1-methyl-4-phenylpyridinium (MPP^+^) ion (Meredith and Rademacher, 2011). However, central dopaminergic neurons were not damaged upon systemic administration of MPP^+^ as it is unable to cross the BBB due to its charge. But, much of the DAergic nigrostriatal pathway was destroyed by direct infusion into the brain. MPP^+^ is selectively taken by the dopaminergic neurons because it is an excellent substrate for the dopamine transporter (DAT) (Meredith and Rademacher, 2011). So we have used MPTP induced mouse model instead of PQ.

Similarly, this study clearly shows that MPTP intoxication produces neuroinflammation induced ROS and RNS overproduction, whereas Mp reduces this ROS and RNS accumulation and downstream proceedings of this cascade. It is well-established that inflammation is the downstream event of oxidative stress. This might be suggested that Mp uses its antioxidative and anti-inflammatory activities to inhibit these oxidative and inflammatory loads. In PD, dopaminergic neurons possess reduced antioxidant ability which makes dopaminergic neurons more susceptible to oxidative stress, as exhibited by the low level of intracellular reduced glutathione. Reduced GSH is one of the main factors responsible for the antioxidant defense, which scavenges free radicals generated in brain tissue (Dringen, 2000). In this study, MPTP injection produces free radicals which creates oxidative damage and is eventually responsible for reduced GSH level along with decreased activity of antioxidant enzyme catalase, while it has also increased the level of lipid peroxidation. This finding in MPTP treated mice is consistent with earlier reports (Cheng et al., 2008; Khan et al., 2010; Lee et al., 2011; Yadav et al., 2013, 2014). Following MPTP injections, treatment with Mp reduced the oxidative injury by decreasing the MDA level along with restoration of GSH level and catalase activity in the nigrostriatum. Recent findings have suggested that oxygen free radical and nitric oxide (NO) play a major role in stress mediated neurodegeneration. In addition, peroxynitrite is formed when NO react with superoxide, which then induces nitration of tyrosine to produce hydroxyl radicals. Therefore, NO along with peroxynitrite may adversely affect neuronal cell death in SN (Yokoyama et al., 2008). Our result exhibits that nitrite level was significantly reduced after Mp treatment which further protected the dopaminergic neurons from NO mediated neurodegeneration, corroborating previous studies (Yokoyama et al., 2008; Yadav et al., 2014).

During the pathogenesis of PD, activation of the glial cells is considered to be a rapid cellular response leading to neuroinflammation (Ghosh et al., 2007; Gordon et al., 2012; Hirsch et al., 2012). Upregulation in the expression of iNOS, ICAM GFAP, TNF-α, and NF-κB after MPTP administration signifies the process of glial activation. Different studies have suggested the presence of activated glial cells in SN and striatum of PD brains (Hirsch et al., 2003; Ouchi et al., 2009). Activation of Glial cells results in NF-κB activation which further triggers the upregulation and release of proinflammatory enzymes iNOS, and proinflammatory cytokine TNF-α in PD (Kim and Joh, 2006; Mosley et al., 2006). Studies done previously suggested that inhibition of glial activation by using some inhibitors prevents MPTP-induced neurotoxicity (Wu et al., 2003; Chung et al., 2011). In our study, inflammatory response due to glial cell activation and dopaminergic neuronal loss is suppressed on treatment with Mp. During neuroinflammation, redox sensitive transcription factor, e.g., NF-κB can be initiated by nitric oxide, ROS and RNS. NF-κB shows activity during neurodegeneration by regulating the expression of different proinflammatory mediators. NF-κB is kept in inhibited form, bounded to inhibitory protein IκB, in the cytoplasm to prevent its nuclear translocation necessary for transcriptional activity. NF-κB regulated transcription of certain proinflammatory genes such as TNF-α, IL-1β, COX-2, enzyme like iNOS, and adhesion molecules (ICAM) occurs only when IκB undergoes phosphorylation and proteolytic degradation resulting in translocation of free NF-κB to the nucleus, where it binds to target DNA elements (gene promoters containing κB binding sites) (Shen et al., 2010). Also, MPTP leads to impairment of the mitochondrial function in PD (Acuna-Castroviejo et al., 2011). As the activity of mitochondrial enzyme complex get inhibited, generation of superoxide anions occurs, which further helps in the process of neuroinflammation by up-regulating NF-κB activation. Thus, it can be suggested that drugs, which can be used in inhibiting the generation of ROS, can be used as neuroprotective agents to protect from the neurotoxin such as MPTP. In this study, Mp has significantly inhibited the production of ROS as well as the activation and translocation of NF-κB. Aqueous extract of Mp treatment improved the normal expression levels of iNOS and GFAP in MPTP treated animals (Yadav et al., 2014). Our aqueous extract of Mp also shows the similar activity as it improved the normal expression levels of iNOS, GFAP, ICAM, and TNF-α in SNpc of MPTP treated animals. Thus, antioxidative and anti-inflammatory drugs can be used effectively as therapeutic agents in the case of neurodegenerative diseases (Jin et al., 2005).

Akt activation helps in the survival of different types of cells including various neuronal types, as reported through various *in vitro* studies. Furthermore, Akt promotes the survival of different neurons by mediating the functions of different neurotrophic factors (Dudek et al., 1997; Crowder and Freeman, 1998; Brunet et al., 2001; Orike et al., 2001; Downward, 2004; Duronio, 2008). Death of cultured neurons occurs when interference with activation of Akt occurs, while, transfection with a constitutively active form of the kinase promotes the survival of neurons in the absence of any other support. Although studies about the Akt signaling is less in the case of survival of non-stressed neurons *in vivo*, it has been more studied in post-natal substantia nigra (SN), the brain area of high relevance in the case of PD (Ries et al., 2009). Dopaminergic neurons numbers get reduced in SN and incidence of apoptotic neurons was doubled due to a dominant negative form of Akt delivered by adeno-associated virus. On the other hand, developmental neuron death in the SN was reduced by transduction of a constitutively active form of Akt. The maintenance of the survival of dopaminergic neurons in SN under basal conditions has been highlighted by these significant findings. Phosphorylation of Akt at Ser473 (Malagelada et al., 2008; Timmons et al., 2009) and Thr308 (Malagelada et al., 2008) is considerably decreased in dopaminergic SN neurons of PD patients when compared with non-PD patients as indicated by the Immunostaining of post-mortem brains. A drop in total Akt staining in such neurons from PD patients was also seen (Timmons et al., 2009). Significantly, in the Ries et al. (2009) study cited above, reduction in number of individual TH^+^ fibers correlated with decreased density of TH^+^ fiber staining in the striatum was seen in the dominant-negative Akt mice model. Our result shows that MPTP inhibits the activation of pAkt1 in the SNpc while Mp treatment overcomes this inhibition, in accordance with the above citation. So, Mp treatment also suggests a possible neuroprotective pathway mediated by pAkt1.

Thus anti-inflammatory pathway especially NF-κB and Akt pathway plays a major role in PD treatment. As MPTP is converted to its active form MPP^+^ inside the astroglial cells, then it is taken by dopaminergic neuron through transporter of dopamine, i.e., DAT (Kostic et al., 1996). These transporters get damaged during MPP^+^ uptake process as number of DAT get reduced in the SN, after MPTP administration (Jakowec et al., 2004). Accordingly, our data also shows that DAT immunoreactivity was reduced in MPTP group while it is considerably improved in Mp treated group. The rate-limiting enzyme in the production of DA, i.e., TH is the marker for the dopaminergic neuron survival. Previous studies explore that TH immunoreactivity was decreased gradually in SN of mice after MPTP treatment. Their findings give important evidence about MPTP-induced neurodegeneration (Kurosaki et al., 2003; Ghosh et al., 2007). The immunohistochemical expression of TH in SNpc region indicates the protective action of Mp in MPTP injected parkinsonian mice, seen in the previous studies too (Kurosaki et al., 2003; Yadav et al., 2014).

The pathophysiology of different diseases such as gout, muscular pain, cancer, arthritis, and other vascular diseases have been seen to be associated with inflammation. Inflammatory symptoms are being treated by using different drugs and natural products (Ahmad et al., 2005). It has been discussed earlier too, that Mp is rich in different constituents such as saponins, tannins, alkaloids, flavonoids in addition to L-DOPA and has been used in the treatment of diseases like fever, muscular pains, spasms and dysmenorrhea in past (Javed et al., 2010) and is thus being tested for its anti-inflammatory activity and the reason behind its traditional use for pain and fever. The results of our present investigation indicates that seed powder of Mp possess significant anti-inflammatory activity by inhibiting the NF-κB and Akt dependent pathways as highlighted by the results of our study.

It is well-established that Mp contain L-DOPA as the primary component (Tripathi and Updhyay, 2001; Tomita-Yokotani et al., 2004; Kasture et al., 2009). The seed of Mp also contains Serotonin and its precursor 5-Hydroxytryptophan (5-HTP), *N,N*-dimethyltryptamine and 5-MeO-dimethyltryptamine (bufotenin) (Ghosal et al., 1971). Different saponins, anthraquinones, flavonoids, terpenoids, cardiac glycosides, and tannins are present in Mp (Agbafor and Nwachukwu, 2011). Data from RP-HPLC analysis of aqueous extract of Mp used in this study revealed a significant peak of L-DOPA correspond to standard L-DOPA peak, Mp also contain various phytochemicals like gallic acid, proanthocyanidin, tannin, quercetin, and phytic acid. Quercetin being one of the major component in our RP-HPLC result, might be showing anti-inflammatory activity. Querctin has shown both antioxidant and antiinflammatory activity as reported earlier by several studies (Haleagrahara et al., 2011; Weng et al., 2012; Costa et al., 2016; Lee et al., 2016). Moreover, other phytochemicals present in Mp might also act in combination to explore its synergistic effect.

## Conclusion

Our study suggests that Mp extract appreciably ameliorate the neuroinflammatory processes and also restore biochemical and behavioral parameters along with TH and DAT immunoreactivity. NF-κB and Akt pathway might be responsible for the underlying mechanism of Mp. The anti-inflammatory activity along with potent anti-oxidant properties shown by Mp extract can be used in treating inflammatory condition in the case of PD.

## Author Contributions

SR, HB, SSS, and WZ: designed and performed the experiments, co-wrote the MS. RP and JJ: performed the HPLC experiments, co-wrote the MS. MG: assisted in the design of the study, statistical analysis, and MS writing. SPS: conceived, designed, directed, and supervised the complete study.

## Conflict of Interest Statement

The authors declare that the research was conducted in the absence of any commercial or financial relationships that could be construed as a potential conflict of interest.

## Supplementary Material

FIGURE S1 Immunofluorescence expression of pAkt1 in SNpc. of CONT, MPTP, and MPTP+Mp mice by using Image J Software at 20x magnification. The MPTP intoxicated PD mice showed significantly decreased expression level of pAkt1 positive cells as compared to control, while on Mp supplementation in PD mice showed significantly elevated expression level of pAkt1 positive cells as compared to MPTP mice. Values are expressed as mean ± SEM of integrated fluorescent value (IFV) (^∗^*p* < 0.05, ^∗∗^*p* < 0.01, *n* = 3).
