# Supplementary figures and images for: Mucuna pruriens Protects against MPTP Intoxicated Neuroinflammation in Parkinson’s Disease through NF-κB/pAKT Signaling Pathways
Source: Front Aging Neurosci. 2017 Dec 19;9:421. doi: 10.3389/fnagi.2017.00421 (PMC5742110; doi:10.3389/fnagi.2017.00421)

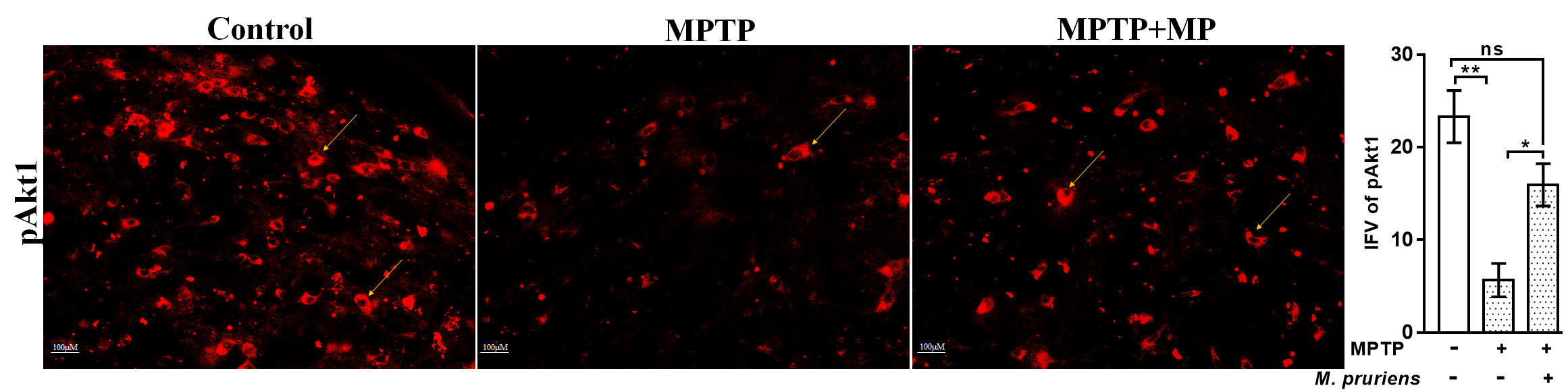

Supplement: FIGURE S1 — Immunofluorescence expression of pAkt1 in SNpc. of CONT, MPTP, and MPTP+Mp mice by using Image J Software at 20x magnification. The MPTP intoxicated PD mice showed significantly decreased expression level of pAkt1 positive cells as compared to control, while on Mp supplementation in PD mice showed significantly elevated expression level of pAkt1 positive cells as compared to MPTP mice. Values are expressed as mean ± SEM of integrated fluorescent value (IFV) (∗p < 0.05, ∗∗p < 0.01, n = 3). [file Image_1.TIF]
